# Analysis of the supply chain and conservation status of sharks (Elasmobranchii: Superorder Selachimorpha) based on fisher knowledge

**DOI:** 10.1371/journal.pone.0193969

**Published:** 2018-03-13

**Authors:** Ana Paula Barbosa Martins, Leonardo Manir Feitosa, Rosangela Paula Lessa, Zafira Silva Almeida, Michelle Heupel, Wagner Macedo Silva, Ligia Tchaicka, Jorge Luiz Silva Nunes

**Affiliations:** 1 Centre for Sustainable Tropical Fisheries and Aquaculture & College of Science and Engineering, James Cook University, Townsville, Qld, Australia; 2 Australian Institute of Marine Science, Townsville, Qld, Australia; 3 CAPES Foundation, Ministry of Education of Brazil, Brasilia–DF, Brazil; 4 Universidade Federal de Pernambuco—Av. Professor Moraes Rego, Cidade Universitária. Recife–PE, Brazil; 5 Universidade Federal Rural de Pernambuco–Rua Dom Manuel de Medeiros, s/n, Dois Irmãos. Recife–PE, Brazil; 6 Universidade Estadual do Maranhão–Cidade Universitária Paulo VI, s/n, Tirirical. São Luís–MA, Brazil; 7 Universidade Federal do Maranhão–Av. dos Portugueses, Bacanga. São Luís–MA. Brazil; Department of Agriculture and Water Resources, AUSTRALIA

## Abstract

Increasing fishing effort has caused declines in shark populations worldwide. Understanding biological and ecological characteristics of sharks is essential to effectively implement management measures, but to fully understand drivers of fishing pressure social factors must be considered through multidisciplinary and integrated approaches. The present study aimed to use fisher and trader knowledge to describe the shark catch and product supply chain in Northeastern Brazil, and evaluate perceptions regarding the regional conservation status of shark species. Non-systematic observations and structured individual interviews were conducted with experienced fishers and traders. The demand and economic value of shark fins has reportedly decreased over the last 10 years while the shark meat trade has increased slightly, including a small increase in the average price per kilogram of meat. Several threatened shark species were reportedly often captured off shore and traded at local markets. This reported and observed harvest breaches current Brazilian environmental laws. Fishing communities are aware of population declines of several shark species, but rarely take action to avoid capture of sharks. The continuing capture of sharks is mainly due to a lack of knowledge of environmental laws, lack of enforcement by responsible authorities, and difficulties encountered by fishers in finding alternative income streams. National and regional conservation measures are immediately required to reduce overfishing on shark populations in Northeastern Brazil. Social and economic improvements for poor fishing communities must also be implemented to achieve sustainable fisheries.

## Introduction

Sharks (Superorder Selachimorpha) play important roles in the health of estuarine, marine, and oceanic food webs worldwide [1, 2, 3, 4, 5]. However, due to a combination of biological and ecological features (i. e. reduced fecundity, late sexual maturation, complex migration patterns), shark species are highly susceptible to anthropogenic pressures [6, 7], such as habitat destruction and overfishing. Over the last 60 years, shark catches by industrial, artisanal, and sport fisheries have increased around the world [8, 9] and sharks are now among the most threatened marine animals [4]. Local consumption of shark meat and high demand for shark fins in Asian markets are major drivers of overexploitation [10, 11]. As a compounding factor, non-reported catch estimates (i. e. misidentified, unrecorded, aggregated, discarded individuals or unmonitored small-scale fisheries) are thought to be 3 or 4 times greater than reported catch [4, 12, 13, 14, 15, 16]. This lack of accurate data could prevent precise estimates of population abundance and fishing pressure which hamper efforts to ensure long-term ecological sustainability [17, 18].

Intense fishing pressure has already caused population declines in coastal and pelagic shark species [4, 19, 20]. In Brazil, efforts to ensure shark conservation have been adopted in the past decade, such as the establishment of minimum size limits, regulation of net mesh sizes, and prohibition of directed shark fisheries and finning practices. Recently, the Brazilian government issued the Ordinance 445/2014 which categorizes elasmobranch species inhabiting Brazilian waters into the following extinction threat categories: Extinct in the Wild (EW), Critically Endangered (CR), Endangered (EN), and Vulnerable (VU). All species listed on this ordinance, except for those classified as VU, are protected and prohibited from capture, transport, storage, handling, processing and trade. Vulnerable species can be harvested only if the Brazilian environmental agency grants authorization. Nevertheless, protected species listed as threatened continue to be caught and harvested throughout the country, especially in the northern region where fishing is mainly artisanal and catch inspections are extremely rare [21]. Despite the introduction of management measures, Brazilian fisheries data are limited, obsolete and rarely consider the social and economic context of the fishers.

To understand the full context of shark fisheries, social factors must be considered through multidisciplinary and integrated studies. Thus, local community motivations to fish sharks should be analyzed [22, 23, 24, 25, 26, 27, 28]. According to the Food and Agriculture Organization of the United Nations (FAO) [29] and Brazilian Ministry of the Environment (MMA) [30], fisher knowledge could be critical for a better understanding of shark fisheries, decision making and directing future research, especially in areas where deficits in fisheries research hinder conservation and management actions. Thus, the present study aimed to use fisher and trader knowledge to: (1) identify the presence of a supply chain of two shark products (meat and fins), (2) describe the supply chain for each product, and (3) qualitatively evaluate fisher and trader perceptions regarding the regional conservation status of shark species in Northeastern Brazil.

## Methods

### Study area

**Fig 1:**
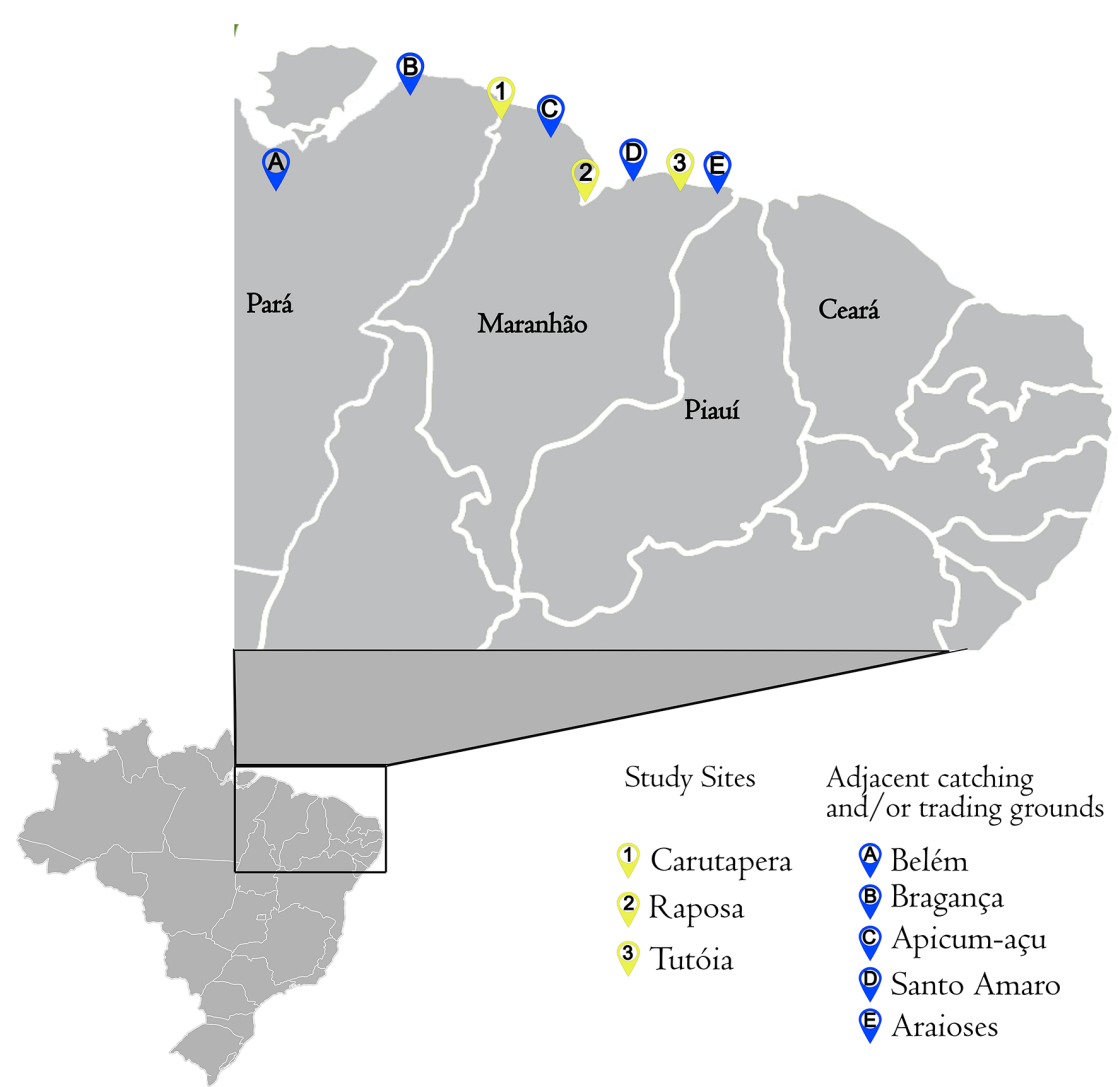
Sampling points in Maranhão, Amazonian Equatorial coast. Visited municipalities: (1) Carutapera (West coast), (2) Raposa (Gulf of Maranhão) and (3) Tutóia (East coast). Adjacent catching and/or trading grounds indicated by fishers: (A) Belém, (B) Bragança, (C) Apicum-Açu, (D) Santo Amaro and (E) Araioses.

Maranhão state has the second largest coastline in Brazil, extending 640 km (Fig 1). The full extent of Maranhão‘s coast is known as an important elasmobranch fishing and conservation area [21, 31]. The coast is geographically divided into three regions based on distinctions in drainage, ocean circulation, and climate conditions: East coast, Gulf of Maranhão, and West coast [32, 33]. To obtain a holistic picture of biotic, abiotic, and socioeconomic features of shark fisheries and commercialization, one sampling area was selected for each region of the Maranhão coast. The selection of sampling areas was based on existing fishing records [31, 34, 35, 36, 37]. For each portion of the coast, municipalities with the highest shark catch rates were chosen as data collection sites: Tutóia (East coast), Raposa (Gulf of Maranhão) and Carutapera (West coast) (Fig 1). Municipalities were visited at least five times between January 2014 and January 2017.

### Data collection and analysis

Data collection occurred through: (1) observations, and (2) structured interviews with individuals involved in shark fisheries and trade. Observations consisted of visits to fishery landing sites and fish markets to record the presence of shark products, investigate the practice of illegal activities, identify traded species and catalog their respective market values. Observations were reported as findings only when verified by more than one trained observer.

Interviews were conducted to evaluate fisher and trader perceptions of shark fisheries, trade (i.e. understanding of final markets and end consumers), and conservation in Maranhão. Each interview was conducted by four experienced interviewers with fishers and traders who work or have worked directly in shark fisheries and/or trade for over 10 years. Although these rigorous criteria reduced the sample size, careful selection of qualified individuals was crucial to accurately record variation in shark fisheries and trade through time. In total, 40 people were interviewed, comprising 32 fishers and 8 traders. All individuals went through an interview process following a structured questionnaire with open-ended questions. No limitations were set for duration or direction of answers during the interview. Questionnaires were divided into four thematic areas to describe socioeconomic drivers of shark meat and fin trade in Maranhão: (1) fisheries and trade, (2) inspection of fishing activities, (3) presence and conservation status of shark species, and (4) causes and perspectives. Interview questions are in S1 File. Questions were designed to be short, clear and with language appropriate to the education level of the interviewees. Questions were repeated if necessary, but no extra comment or explanation was provided to reduce the chance of biasing answers.

Information regarding inspection activities was requested and obtained from the Brazilian Institute of Environment and Renewable Natural Resources (IBAMA)–the federal agency responsible for enforcement of environmental laws, including fishing activities. Species identification was carried out through comparison of common names provided by interviewees with specimen observations at landing or trading sites by trained observers. Identification guides [38, 39, 40] were also used by the observers as a support tool to ensure correct identification. Nevertheless, identification to species level was not possible for *Sphyrna* spp. due to the loss of identifying features on carcasses. Since this study spanned several years, conversion of the Brazilian currency (Real) to U.S. dollars was calculated using annual mean exchange values. Prior to conducting interviews this research protocol was submitted to the research ethics committee at Universidade Federal do Maranhão. The committee determined that ethical clearances were not required. Nevertheless, the study was conducted according to the principles in the Declaration of Helsinki and the identity of all interviewees was kept anonymous.

## Results

### Fisheries and trade

Interviews were conducted with 19 individuals in Tutoia, 10 in Raposa and 11 in Carutapera. Average age of interviewees was 45 years old, with an average of 24 years fishing experience. Most interviewees (61%) had been fishing since childhood. Interviews indicated shark catches have occurred in Maranhão for at least 60 years. Captures have increased in the past four decades when fishers from other states of Brazil, encouraged by Asian traders, moved to the region to initiate fin trade. Observations revealed fishing activity in the region matched Barreto et al. [21] and Almeida [41]’s previous descriptions that characterized fisheries in Maranhão as mainly artisanal and conducted by individuals or family groups. Although fishing characteristics varied between municipalities, most fishers rely on motor or sail vessels, rarely use GPS and, in many instances, have limited safety equipment. Most vessels are small to medium sized (on average 5–10 m), use both gill net and longlines to fish, operate in shallow areas and have limited crew. For these reasons, fishing efforts are mainly concentrated in estuaries, bays, and shallow coastal waters. On average, fishermen from Tutoia spend more time fishing than those from Raposa and Carutapera. As a general rule, larger vessels with more powerful engines and crews fish longer than smaller vessels (Table 1).

**Table 1:** Description of vessel and fishing features used in each region of the Maranhão coast. Information derived from observations and interviews.

Municipality	Vessel size (m)	Engine strength (HP)	Fishing gear	Gear soak time (h)	Crew members	Fishing period (days)	Mean depth of catch (m)
Tutóia	10–13	18–75	Gill net and long line	2–8	4–6	15–20	50
Raposa	5–9	18–75	Long line	6–12	4–10	1–5	30
Carutapera	2–13	18–75	Gill net and long line	2–8	4	1–25	50

### Supply chain

Supply chain analysis evaluated the linkage of activities from fishing to processing and distribution of shark meat and fins. Sharks landed in Maranhão ports were not always captured in maritime zones belonging to the municipality or even to the state. Localities such as Canárias (Araioses-MA), Apicum-Açu (MA) and Travosa (Santo Amaro-MA), the cities of Bragança and Belém, in the state of Pará, as well as the states of Piauí and Ceará were also identified as important catching and/or trading grounds (Fig 1). Maranhão’s landing points were used due to their strategic location between the north and northeast coast of Brazil and based on high local demand for shark meat and fins.

**Fig 2:**
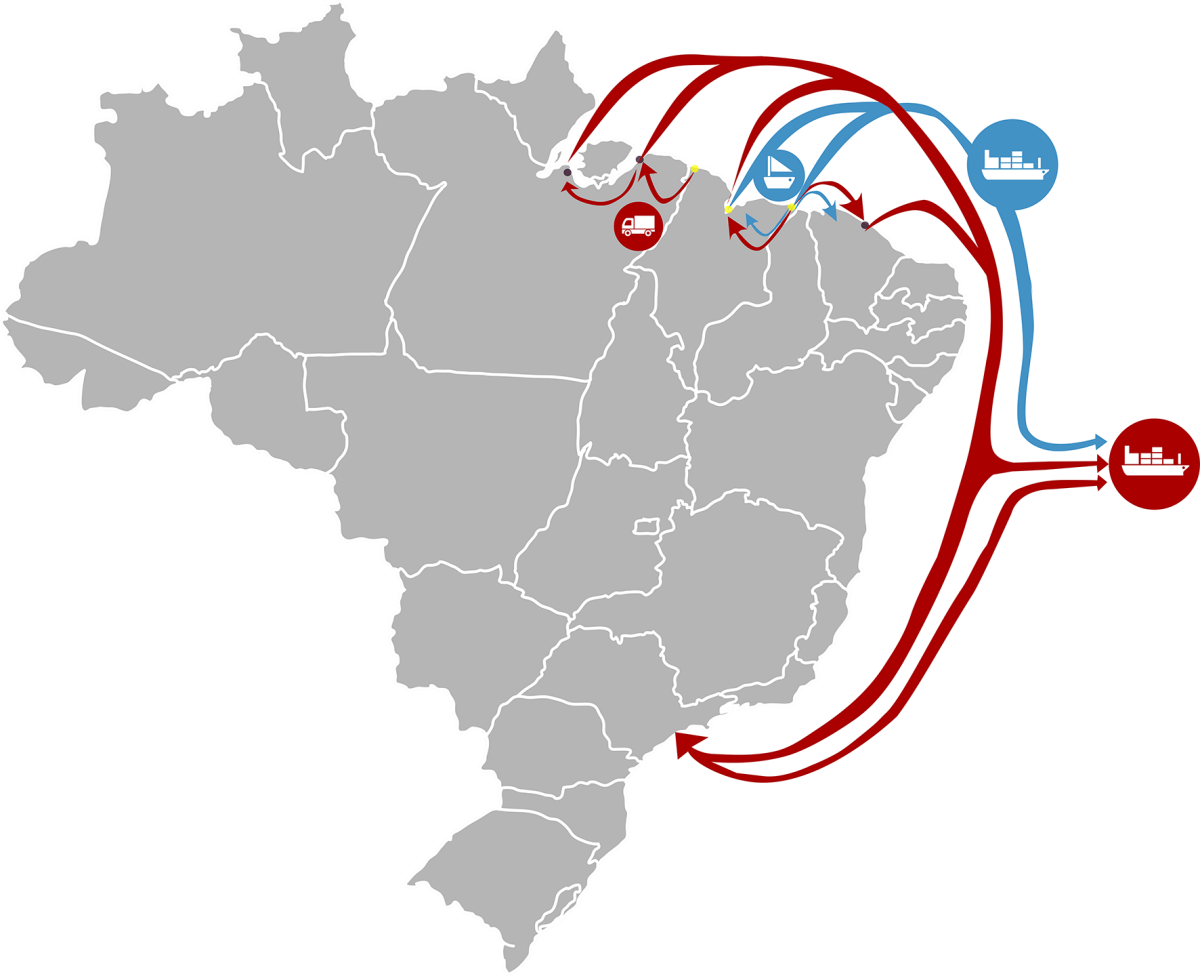
Supply chain of shark fin trade in Maranhão state over the past 15 years. The red line depicts fin trade before regulations (before 2010) and the blue line depicts the fin trade after introduction of regulation (after 2010). Icons reflect the type of shipping transportation of fins. Sample sites for this study are marked as yellow dots. Cities outside Maranhão state participating in the supply chain are marked as black dots.

**Fig 3:**
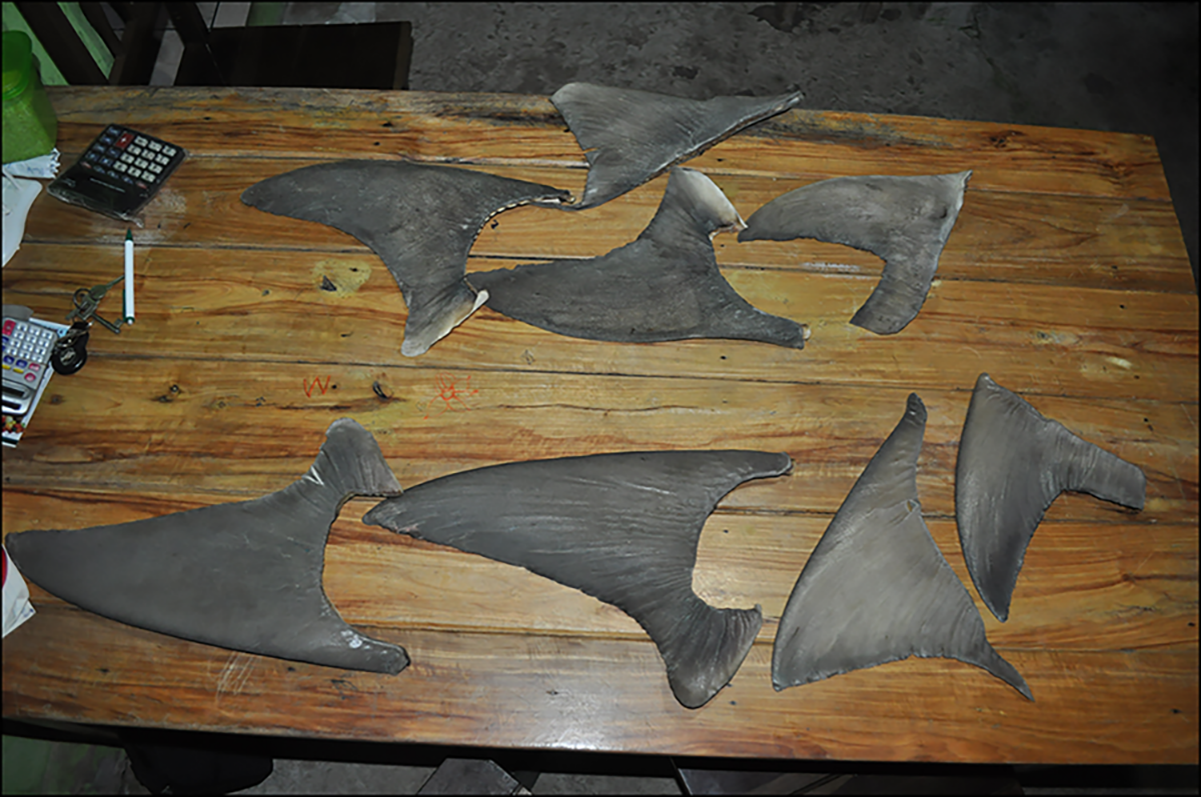
Dried shark fins over 35 cm length.

Shark fin trade was reportedly the most profitable activity in all regions from 1990 to 2010. After removal, fins were sun dried and sent to major ports, such as the port of Santos, Sao Paulo state in southeast Brazil, to facilitate export to Hong Kong. Each kg of fins was valued from US$ 235.00 in Raposa to US$ 353.00 in both Carutapera and Tutoia. Recently, shark fin trade has declined in some areas and disappeared in others (Fig 2). In Tutóia, for example, only fins 35 cm or larger continue to be traded (e.g. *Carcharhinus leucas* Müller & Henle, 1839 and *Sphyrna* spp.) (Fig 3), but, according to the interviews, the value has decreased by 85% since 2005. Interviewees reported increased inspections at international ports, fear of product seizure, and the consequent lack of interest by Asian buyers as the main causes for decreasing trade. According to interviewees, it is no longer possible to rely on shark fin trade as a source of income in Maranhão. Some fishers changed their focus to more profitable species and activities, such as trading shark cartilage and acoupa weakfish (*Cynoscion acoupa* Lacepède 1801) swim bladders.

**Fig 4:**
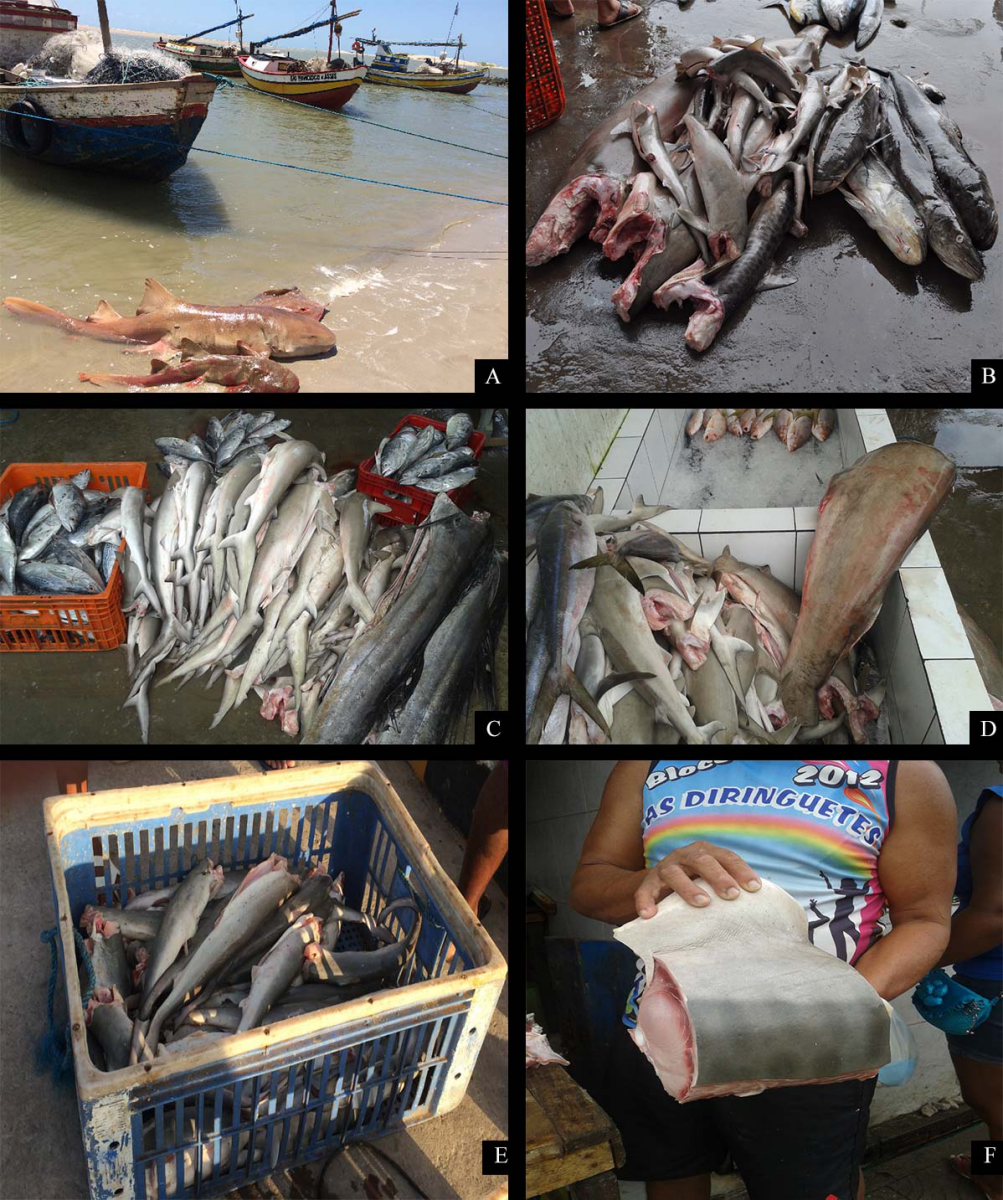
Shark meat trade in Maranhão. (A) Two *Ginglymostoma cirratum* specimens landed whole; (B), (C), (D) landed carcasses with fins attached; (E) landed carcasses without fins attached; (F) trade of fresh *Galeocerdo cuvier* meat.

The east coast of Maranhão had the highest commercial sale of shark meat. Currently, fresh and salted meat are the main shark products traded in the region (Fig 4). However, supply has decreased and shark products are not abundant items in local fish markets. The value of fresh meat has increased from US$ 1.20 to US$ 4 per kg over the last decade. According to interviewees, most of the shark meat available was consumed locally, but when the amount available exceeds local consumption (average of 2000 kg), traders sell mostly to Camocim, Ceará state (237 km from Tutoia). Consumption of shark meat in the Gulf of Maranhão was mainly local, although a small amount may be sent to markets in adjacent communities. The scenario on the west coast was slightly different, since shark catch was low and only capable of meeting local demand.

### Inspection of fishing activities

IBAMA carries out inspections throughout Maranhão’s coast. Fishers reported varied experience with inspections and interactions with IBAMA. Only 12.5% of interviewees reported that inspections were performed at least three times per year and individuals informed about illegal practices being conducted such as violations of size limits, use of illegal methods and capture of protected species. However, 88.5% of respondents claimed inspections and IBAMA interactions with the fishing community were rare or absent, resulting in a lack of communication. The Carutapera landing point was an exception. Inspections were reportedly consistent and continuous in the area, especially on the border between Maranhão and Pará, but were performed by Pará state’s Department of Agriculture (SEFA-PA) rather than IBAMA. SEFA-PA was indicated as responsible for product seizure and fines for individuals caught transporting shark products (especially fins) across the border without proper documentation. According to interviewees, SEFA-PA inspections were one of the causes of the drastic decline in shark fin trade in western Maranhão.

All interviewees demonstrated knowledge regarding shark finning prohibitions in Brazil. In contrast, 63.1% had no knowledge regarding protected status of shark species along the Brazilian coast. Consequently, none of the interviewed fishers adopted strategies to minimize or avoid catching threatened shark species.

### Presence and conservation status

Seven species (*Carcharhinus acronotus*, *C*. *leucas*, *C*. *limbatus*, *C*. *porosus*, *Galeocerdo cuvier*, *Ginglymostoma cirratum*, and *Rhizoprionodon porosus*), and a group of species (*Sphyrna* spp.) were reported by interviewees as common catches and observed in fishing markets. *Sphyrna* spp. and *G*. *cirratum* were reported as the most frequently captured species. Capture and trade of *C*. *porosus* (Critically Endangered/ Ordinance 445/2014), *G*. *cirratum* (Vulnerable/ Ordinance 445/2014) and *Sphyrna* spp. (Endangered/Critically Endangered/ Ordinance 445/2014), even if incidentally caught, is prohibited by Brazilian law. Reported catch of these species and their presence in markets is direct evidence of violation of Brazilian law.

The most frequently found species in the local market, *R*. *porosus*, had the highest demand in the local community. Shark availability in the fishery was reported throughout the entire year, but more frequent during the rainy season. All interviewees highlighted that shark catches have been declining over the past two decades, even though fishing effort has increased. Species easily caught in the 1980s and 1990s such as *C*. *porosus* (Ranzani, 1839), *Isogomphodon oxyrhynchus* (Müller & Henle, 1839) and *C*. *limbatus* (Müller & Henle, 1839) are now scarce and only found in deeper areas further from the coast. In general, fisher perception of shark conservation status corroborates recent evaluations by Brazilian environmental agencies. Fishers indicated, for example, that *C*. *porosus* is the species at greatest risk of extinction in Maranhão. The only exception was *G*. *cirratum*, for which catches were considered common during the rainy season.

### Causes and future perspectives

Overfishing was reported by 86.4% of interviewees as the main cause for shark population declines along Maranhão’s coast. There was common agreement that commercial exploitation could cause shark population collapses soon. In addition, increases in fishing activities have occurred under weak regulation enforcement. Due to lack of education and enforcement, fishers continue to operate indiscriminately, maximizing profits and jeopardizing future stocks of both sharks and heavily consumed teleost fishes. Indeed, interviewees reported that it is increasingly difficult to catch sharks, even to meet local demand.

## Discussion

Based on interviews it is apparent that fishers along the Maranhão coast are aware of shark population declines, but catch and trade of products continues. Numerous violations to Brazilian environmental laws were observed and reported in all sampled locations (e.g. fisheries targeting sharks, capture and sale of protected species). It is clear that existing shark management and conservation strategies are not achieving the expected results and shark populations along Maranhão’s coast continue to decline. Our results reinforce the need for actions to reduce shark overexploitation in Brazil.

### Fisheries, trade and supply chain

Currently, shark catches along the Maranhão coast are mainly incidental, although targeted capture does occur for local consumption. In the last 45 years, shark catches have increased unsustainably despite efforts by state and federal governments to maintain sustainable exploitation [42, 43, 44, 45, 46]. According to Almeida et al. [47], increasing acceptance of shark meat as a food source and depletion of other traditionally exploited fish resources (e.g. *Cynoscion acoupa*, *Scomberomorus brasiliensis*) are important factors in the intensification of shark fishing since the 1970s. However, increased shark catches were mainly related to the high value of shark fins [12, 13, 14, 47, 48, 49, 50]. High demand for shark fins brought fishers and traders from different parts of Brazil to Maranhão. Fleet size and fishing effort increased, but the fishery structure remained predominantly artisanal [41]. Between 1970 and 1980 elasmobranchs comprised over 60% of the total catch in Maranhão by weight [34], indicating the importance of shark products to the economy.

Between 1970 and the mid-2000s, fins represented the most profitable shark product in the state, followed by meat [36, 41, 47]. Since 2010 this pattern has slowly changed. After initiation of international and national prohibitions, sale of shark fins in Maranhão decreased. Since inspections on fishing and landing points were reported as rare or non-existent in two of three sampling sites, declines in shark fin trade in Maranhão are more likely the result of decreasing demand for shark fins in Asian markets than enactment of national regulations. These results follow the pattern reported by Shea & To [51], who observed a decline in demand and consumption of shark fins in Chinese restaurants after public concerns were raised.

Shark meat imports in Brazil, including Maranhão, have substantially increased since 1990 [21]. In the Gulf of Maranhão and along the west coast, shark catches are mainly incidental and landings are sporadic. However, fisheries focused on elasmobranchs were observed on the east coast. Among the areas analyzed, the east coast reported higher numbers of shark specimens and species traded. This occurred due to the greater sale of shark meat and the limited, but still evident, demand for shark fins. Currently, meat represents the most profitable shark product in the state. Anti-finning regulations have encouraged full utilization of shark carcasses, which were previously discarded [10]. Smaller species, regionally called “*cação*”, are widely consumed by local communities due to the softness of the meat and low cost (US$ 1.50–2.00, on average). The price per kg of shark meat tends to decrease for larger species. Dislike of large shark meat appears to stem from bias based on fear of large sharks [36] and the high concentration of ammonia, and consequent strong smell of the meat [52, 53]. Only two exceptions were observed, *Sphyrna lewini* (Griffith & Smith, 1834) and *C*. *limbatus*, which were widely reported in local markets and consumed.

### Inspection and oversight

Many problems inherent to the inspection of shark fisheries and trade were reported and/or observed in this study. First, was the apparent limited number of agents and surveillance boats. The extensive length of the Maranhão coast requires a large number of agents to maintain monitoring. According to IBAMA-Maranhão (personal communication), the present number of field agents is insufficient for comprehensive landing point inspection. Moreover, IBAMA-Maranhão is not equipped with vessels, thus open sea interventions only occur when conducted with other monitoring agencies. This results in very limited capacity to monitor and restrict shark catches and product sale.

The frequency and intensity of inspections was also limited. According to IBAMA-Maranhão (personal communication), three inspections are performed per year along the Maranhão coast. However, the frequency and intensity of inspections are not the same for all sampling sites. In Raposa, the closest point to Maranhão’s capital and where IBAMA’s head office is located, inspections are not common, but have been reported. In regions farther from the capital, Tutoia and Carutapera, inspections are reportedly rare or nonexistent.

Finally, the lack of information regarding shark fisheries activities and trade impedes management. In all visited localities, communities were aware of the shark finning prohibition in Brazilian coastal waters. However, only a small number of fishers were able to name any species under threat of extinction and protected by law. IBAMA-Maranhão reported that information about prohibitions and protection is sent to community leaders, including fisher and trade unions. Nevertheless, it is evident this information is not communicated to individuals involved in fishing, which often results in illegal fishing. Communication between inspection agencies and community leaders is urgently needed. Official agencies and NGOs acting in the area are expected to supply information to fishers and communities. Likewise, community leaders and unions are expected to disseminate information to fishers and traders. If this observed lack of communication persists, shark conservation will not be achieved.

### Presence and conservation status

Reports indicate that the number of sharks caught has declined when compared to previous decades at all sampling sites. Species commonly caught a few decades ago, such as *C*. *porosus* [31, 34, 35, 54] and *I*. *oxyrhynchus* [32, 55], are now rarely or not encountered. In fact, shark catches were much higher during the 1980s when approximately 60% of the fisheries production in Maranhão state was composed of elasmobranchs [31, 34, 42]. Since there are no recent catch records for the state, a direct comparison is impossible. However, several species previously common are now reported as rare by experienced fishers and have been classified as threatened with extinction in the region (i. e. *C*. *porosus*, *I*. *oxyrhynchus*, *Sphyrna tudes*, *S*. *tiburo*, *S*. *mokarran*, *S*. *lewini*).

One exception to declining trends was *G*. *cirratum*, which is now rare or locally extinct from other areas of occurrence along the Brazilian coast [56]. However, in this study, *G*. *cirratum* catches were frequently reported in two of the three sampling areas (east coast and Gulf of Maranhão). In addition, 100% of interviewees from the Gulf of Maranhão considered *G*. *cirratum* a highly abundant species and difficult to avoid capturing throughout the rainy season. This differs from patterns found in other Brazilian regions, where the species is now rare [54, 57] and seasonal variation in abundance has not been observed [58]. Based on Almeida et al. [36], strong winds and rough seas during the dry season reduce fishing effort on Maranhão’s coast, which could explain reduced capture of *G*. *cirratum* and other species during this period. However, more data are needed to explain seasonal variation in *G*. *cirratum* abundance at Maranhão and evaluate whether this high local abundance of *G*. *cirratum* represents one of the last strongholds of the species in Brazil.

Capture of *Sphyrna* spp. was also frequent in Maranhão [32, 35, 47, 55]. Capture and retention of these species violates national legislation (Ordinance n 445 of the Brazilian Environmental Ministry/Ministry of Fishing and Aquaculture, Dec. 17^th^, 2014) that prohibits capture, transport, and trade of *Sphyrna* species in Brazilian waters. Fishing pressure on *Sphyrna* species remains high and occurs mainly due to the consistently high value of their fins [36, 47, 49]. Declines in hammerhead shark populations are widespread and apparent based on interview results and corroborating studies conducted in different parts of the world [59, 60, 61, 62], which highlight the vulnerability of these species. These findings highlight the need for management and conservation strategies directed toward this group along the Maranhão coast to help maintain local and regional populations.

### Causes and perspectives

Shark products are an important source of income and protein around the world [63, 64], including Maranhão. Thus, social and economic realities of fishers and their work conditions are important drivers of shark exploitation [41]. Many respondents from both western and eastern regions live in unsafe housing built of mud and mangrove wood covered with palm leaves. Fishers have little or no education, with most not having completed elementary school. Health and sanitation conditions are also precarious, with many fishing villages lacking potable water [41]. In addition, profit sharing from fishing activities is often unfair. Those responsible for the vessel and fishing gear regularly get the majority of the profit [50, 65]. In contrast, fishers receive only a small percentage of the profit and, in some cases, are paid in product (fish). This traditional distribution of income triggers an inversely proportional relationship between the search for resources and environmental awareness. In this scenario, fishers are easily compelled to ignore conservation laws and exploit fish to increase profits in order to improve quality of life for themselves and their relatives. In addition, as observed by Cedric [28] in Tanzania, it is very difficult to implement effective management measures when fishing communities lack a proper understanding of the resource system. In our study, although fishers have reported shark population declines, they do not seem to have a good understanding of the system and current regulations. This is not encouraging, especially as the Brazilian Institute of Geography and Statistics and MMA consider Maranhão’s coast an extremely important area for elasmobranch conservation. It is the responsibility of federal and state governments to take steps to reverse this scenario through effective implementation of the National Plan of Action for Sharks (NPOA) guidelines, fisher education, communication and enforcement of legislation.

## Conclusion

This study identified and described the supply chain of shark products, in conjunction with fisher and trader perceptions of regional conservation status of shark species, along the Maranhão coast in Brazil. Results revealed that fishing communities are aware of shark population declines, but rarely act to maintain and conserve sharks. This disregard for species status is compounded by a lack of knowledge about laws, lack of enforcement by responsible authorities, and difficult socioeconomic issues encountered by fishers and traders. This study provides evidence of the intricate networks involved in the consumption of shark products in poor communities, reinforcing the fact that social factors must be analyzed to understand the full context of shark fisheries. In this scenario, we reinforce the need for long-term management strategies that aim to enhance ecological and economic sustainability along the Maranhão coast. Furthermore, we provide key information to enhance effectiveness of environmental agencies and thus decrease the occurrence of illegal fishing activities in the area. We highly recommend a review of the existing fisheries policies that currently neglect social and economic factors, and more importantly their effective implementation and monitoring. To improve the population status of sharks along the Maranhão coast and elsewhere in Brazil, improved national and regional measures must be put in practice immediately. This study identifies the composition of people and markets in the shark product supply chain, reasons why people interact, inefficiencies in the system and, finally, provides guidance to enhance current resource management strategies.

## Supporting information

S1 File Interview questions. (DOCX)

S2 File Consumption of shark products: How to achieve sustainable fisheries in poor communities? Developed online: https://magic.piktochart.
(TIF)
