# Supplementary material for: Analysis of the supply chain and conservation status of sharks (Elasmobranchii: Superorder Selachimorpha) based on fisher knowledge
Source: PLoS One. 2018 Mar 13;13(3):e0193969. doi: 10.1371/journal.pone.0193969 (PMC5849302; doi:10.1371/journal.pone.0193969)
Supplement: S1 File — (DOCX) [file pone.0193969.s001.docx]

QUESTIONNAIRE

- How long has shark fishing been occurring in this area?

- Where does it happen? Mangrove forest, river mouth, open ocean?

- How many people are involved? (How many work with you? Are they family members? Besides you, how many more are colleagues?)

- What kind of vessel do you use to fish sharks? (Canoe? Propeller? Central propeller? What’s the size? What’s the power? How many cilinders?)

- What is the main fishing gear used? (How do you fish? What kind of gear do you use the most?)

- What is the average fishing duration? (What time do you go fishing and come back? How much time do you stay in the water? How long do you wait to verify and retrieve sharks from fish-weirs?).

- What are the main species captured? (What kind of shark is the most common?)

- Is there a size limit to fish? (What is the maximum size of a shark your boat can withstand?)

- On average, how many sharks are landed daily? (How many animals do you catch per day?).

- Which animal parts are traded? (What do you sell from the shark? The whole animal? Fins? Meat? Fresh, frozen or salted?).

- Most shark harvested is sold locally or exported? (Do you sell in the local market? The capital or do you send it away?)

- Who buys? (Local folks? Does somebody comes to buy? Do they always buy it? How do they come to buy? Where do they take it?).

- Has the price of the shark meat change? (What about the price? It used to be more expensive? Has something changed now?).

- How does the fin trade occur? (Who buys? Where are they taken? How much it used to cost? How much does it cost now? Do you know if the price went down? Why did it decrease?).

- Are there fishing restrictions? (Are you aware of any fishing restriction? Pregnant female, type of gill net, endangered species, reproductive period, small or large sized sharks?).

- Do inspections occurr? (Did somebody ever come here disturb you due to shark harvesting? People from IBAMA, the State, City administration? How often do they show up?).

- Are there clarifications about rules and prohibitions by the agencies responsible for inspections? (Did somebody ever explained to you the reason for fishing prohibitions? Did someone ever tell you that fishing a certain species or using a certain type of net is prohibited?).

- Do you know which species are protected by law? (Do you know examples of shark species that cannot be harvested?).

- Are there strategies to avoid capturing said species? (What do you do when you catch a species that cannot be fished?).

- What was the maximum size you’ve ever found? (What was the biggest shark you ever caught? What size and species was it?).

- Do you catch sharks all year? Is there a period they are more abundant?

- Is there knowledge of the reproductive periods of any species? (When do females get pregnant? Which size are they when they have pups? How many pups? How much time do they stay with the pups before giving birth?)

- Do you know any nursery areas? (Where do you catch the pups?)

- Do you captured males or females? (Do you catch more males than females or so so?).

- What is the amplitude of the birthing area? (Do pregnant females stay in the same place? Do they get separated from the males? And the pups? Do they change places after giving birth?).

- What is the average gestation period? (How long do you think a female stays pregnant?).

- Are there indications of population reduction for any species? If so, which one(s)? (Do you - know about any kind of shark you rarely see now? What was the last time you caught one of those? Do you know why they disappeared?).

- What is the ecological situation of this species? (Would you say this species is doing fine, more or less, it is bad, or really bad?).

- What is the distribution of this species? (Where do you catch it more often? How many kilometers?).

- Is there indications of degradation in the region over the last 10 years? (Do you know if there has been any kind of destruction in the mangrove, water pollution or major constructions that have damaged fishing here?).

- In your opinion what is the scenario for the next years? (What do you think is going to happen to this species over the next 10 years?).
