# Supplementary figures and images for: Analysis of the supply chain and conservation status of sharks (Elasmobranchii: Superorder Selachimorpha) based on fisher knowledge
Source: PLoS One. 2018 Mar 13;13(3):e0193969. doi: 10.1371/journal.pone.0193969 (PMC5849302; doi:10.1371/journal.pone.0193969)

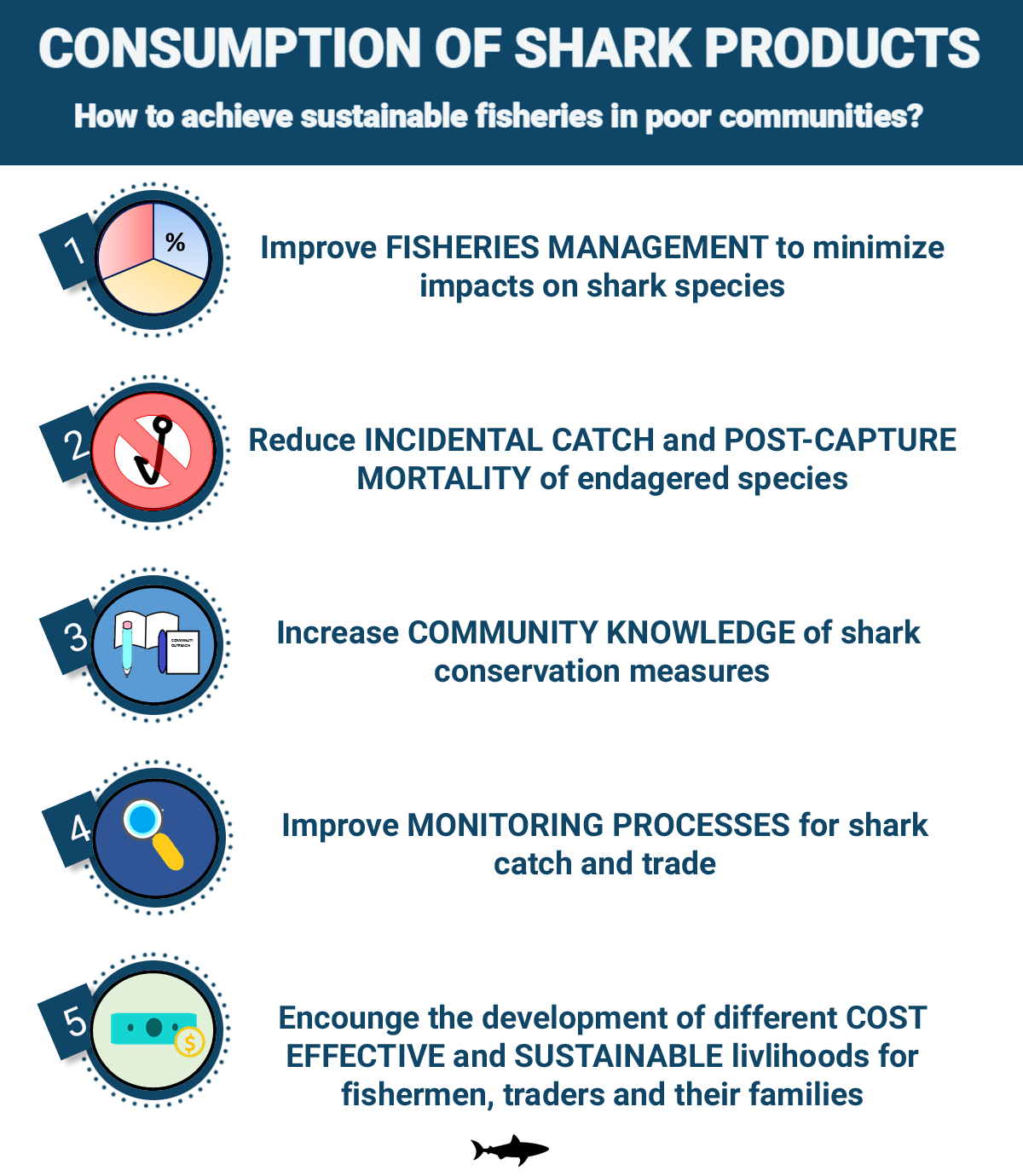

Supplement: S2 File — Developed online: https://magic.piktochart. (TIF) [file pone.0193969.s002.tif]
